# Sulfur-doped silicon oxycarbide by facile pyrolysis process as an outstanding stable performance lithium-ion battery anode†

**DOI:** 10.1039/d4ra04608k

**Published:** 2024-09-20

**Authors:** Jungjin Park, Won Young An, Keunho Lee, Seungman Park, Minjun Bae, Seon Jae Hwang, Hwichan Hong, Yonghwan Kim, Taehyun Yoo, Dohyeong Kim, Jong Min Kim, Yuanzhe Piao

**Affiliations:** a Graduate School of Convergence Science and Technology, Seoul National University 145 Gwanggyo-ro, Yeongtong-gu Suwon-Si Gyeonggi-do 16229 Republic of Korea parkat9@snu.ac.kr; b Advanced Institutes of Convergence Technology 145 Gwanggyo-ro, Yeongtong-gu Suwon-si Gyeonggi-do 16229 Republic of Korea; c Samsung Electro-Mechanics 150, Maeyeong-ro, Yeongtong-gu Suwon-si Gyeonggi-do 16674 Republic of Korea vitamin66@snu.ac.kr

## Abstract

Silicon oxycarbide (SiOC) is drawing significant attention as a potential anode material for lithium-ion batteries due to its remarkable cycle life and the distinctive Si–O–C hybrid bonding within its structure. However, a notable drawback of SiOC-based electrodes is their poor electrical conductivity. In this study, we synthesized sulfur-doped silicon oxycarbide (S-SiOC) *via* facile one-pot pyrolysis from a mixture of commercial silicone oil with 1-dodecanethiol. Upon testing the S-SiOC electrode materials, we observed significant attributes, including an outstanding specific capacity (650 mA h g^−1^ at 1 A g^−1^), exceptional capacity retention (89.2% after 2000 cycles at 1 A g^−1^), and substantial potential for high mass loading of active materials (up to 2.2 mg cm^−2^). Sulfur doping led to enhanced diffusivity of lithium ions, as investigated through cyclic voltammetry (CV) and galvanostatic intermittent titration technique (GITT) tests. Consequently, this sulfur-doped silicon oxycarbide, exhibiting excellent electrochemical performance, holds promising potential as an anode material for lithium-ion batteries.

## Abstract 2

Silicon oxycarbide (SiOC) is drawing significant attention as a potential anode material for lithium-ion batteries due to its remarkable cycle life and the distinctive Si–O–C hybrid bonding within its structure.

## Graphical abstract

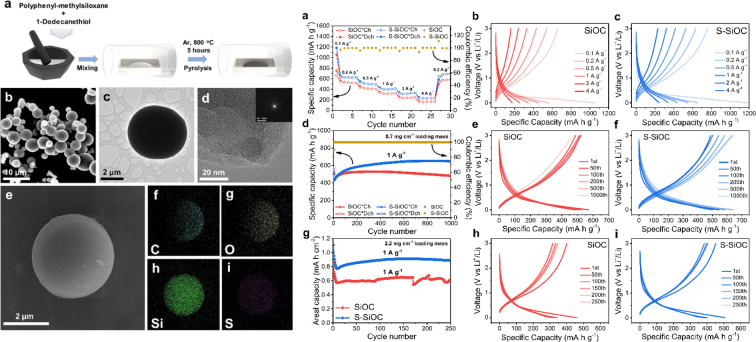

## 1. Introduction

Lithium-ion batteries (LIBs), renowned for their high energy density, power density, and exceptional cycling performance, are one of the most potent energy storage systems (ESS). These batteries have been extensively explored for diverse applications, including portable devices and electric vehicles (EVs).^1–4^ Graphite has seen widespread adoption and commercialization as an anode material in the battery domain due to factors such as its low operating potential *vs.* Li^+^/Li, limited volumetric change during cycling, and cost-effectiveness.^4,5^ Nevertheless, with the rising demand for increasingly advanced electronics, the limitations of graphite have become evident, primarily due to its low theoretical capacity of 372 mA h g^−1^. As a result, a plethora of studies have been undertaken to explore alternative candidates. The silicon-based anode has emerged as one of the most enticing materials, boasting a theoretical capacity surpassing 3579 mA h g^−1^. Nonetheless, the substantial volume change observed during cycling, reaching up to 400%, remains a significant challenge as it can lead to particle fractures and decreased electrical contact. To address this issue, several innovative strategies have been implemented in the development of Si/C composite materials.^6–8^ However, despite these efforts, the problem of capacity fading in Si/C composite materials still persists, primarily attributed to structural breakdown during cycling.

In this context, silicon oxycarbide (SiOC) ceramics have recently garnered attention as potential anode materials due to their potential to replace carbonaceous and silicon-based anodes. SiOC exhibits thermal and structural stability, good cycle performance, a simple synthesis process, and an impressive reversible capacity of approximately 600 mA h g^−1^, attributed to its distinctive structure.^9^ SiOC ceramics can be represented by SiO_*x*_C_4−*x*_ + *y*C_free_ (0 ≤ *x* ≤ 4), wherein SiO_*x*_C_4−*x*_ refers to the Si–O–C glass phase and C_free_ corresponds to the segregated free carbon phase. However, despite these promising features, SiOC-based electrodes still face some significant challenges.^10^ One of the primary issues with SiOC electrodes is their low initial coulombic efficiency, which means that a significant amount of the initial lithium introduced during the first cycle is not efficiently utilized and results in inefficiencies in energy storage. Additionally, SiOC electrodes often exhibit voltage hysteresis, where the voltage during charge and discharge does not align precisely, resulting in energy losses and reduced overall efficiency. Another significant challenge is the inferior electrical conductivity of SiOC electrodes. This limitation is primarily due to their inherently electrically insulating characteristics and limited ionic transport capabilities.^9,11,12^ SiOC materials typically have lower conductivities, particularly for LIB applications, because of the open structure of free carbon and the lack of a sufficient volume fraction of conductive carbon necessary to form a continuous conducting network.^13^ This low conductivity can lead to poor rate capability and hinder their overall performance in LIBs.

In response to these challenges, diverse strategies have been employed to enhance SiOC-based electrodes. To augment electrical conductivity, various types of carbonaceous materials, including carbon nanotubes,^11,14^ carbon nanofibers,^15^ and graphene,^16–18^ have been integrated into composite structures with SiOC. Moreover, the creation of hybrid anodes by combining SiOC with metals has been explored as a viable route to enhance electrochemical performance.^19,20^ Kaspar *et al.* proposed a stable SiOC/Sn nanocomposite achieved through chemical modifications of distinct polysiloxane precursors with tin(ii) acetate, followed by an annealing pyrolysis process at 1000 °C. The resulting SiOC/Sn nanocomposite anode demonstrated a capacity of 562 mA h g^−1^ at a current density of 74 mA g^−1^, exhibiting minimal capacity fading over 20 cycles, and retaining a capacity of 133 mA h g^−1^ even under high current rates of 744 mA g^−1^.^20^ Doping also presents a noteworthy approach to achieve enhanced electrical conductivity and improved electrochemical activity. The introduction of heteroatoms (such as nitrogen, sulfur, and phosphorus) can establish active sites and modify electronic structures.^21,22^ In the realm of energy storage systems, various carbonaceous materials used as anode components, such as graphene, carbon nanotubes, and porous carbon, have utilized heteroatom doping to enhance their performance.^23–29^ Among these, sulfur has gained attention as a promising dopant for electrochemical applications, owing to its smaller atomic radius and electronegativity.^30,31^ Additionally, sulfur introduces supplementary electron transfer pathways *via* C–S–C bonding.^32^ Despite the various advantages of sulfur doping in boosting electrochemical performance, the utilization of H_2_S gas as a sulfur source in doping procedures raises safety concerns due to its inherent toxicity and flammability.^33,34^

In this study, we present, for the first time, sulfur-doped silicon oxycarbide (S-SiOC) as a promising anode material for lithium-ion batteries. We employed a novel sulfur doping approach for silicon oxycarbide by utilizing a facile pyrolysis process involving commercial silicone oil and 1-dodecanethiol under an inert atmosphere. Importantly, this sulfur doping was achieved without the use of H_2_S gas. Through this process, sulfur atoms were successfully incorporated into the silicon oxycarbide lattice, forming covalent bonds with carbon atoms, and generating a partially heterocyclic structure. This strategic sulfur doping not only facilitated efficient electron transfer by creating additional pathways through C–S–C bonding but also resulted in increased (002) interlayer spacing and the provision of extra active sites, thereby enhancing Li^+^ accommodation. As a result of these modifications, the prepared S-SiOC material exhibited remarkable electrochemical performance, substantial cycle stability, and exceptional rate capability. Impressively, even after 1000 cycles, the electrode maintained a capacity of 650 mA h g^−1^ without any observable capacity fading. Notably, the electrode displayed an impressive capacity retention rate of 89.2% after 2000 cycles at 1 A g^−1^. Moreover, during 250 cycles at 1 A g^−1^ (at a mass loading of 2.2 mg cm^−2^), the electrode demonstrated an areal capacity exceeding 0.88 mA h cm^−2^.

## 2. Experimental section

### 2.1. Materials

Silicone oil (AP1000, polyphenyl-methylsiloxane) was purchased from Aldrich Chemical Co., and 1-dodecanethiol (>98.5%) was purchased from Samchun Co. All materials were handled without any purification.

### 2.2. Preparation of SiOC and S-SiOC

Both bare SiOC and S-SiOC were synthesized using a facile one-pot pyrolysis involving liquid precursors. To prepare bare SiOC, 4 g of silicone oil were directly added to an alumina boat without any pretreatment. The oil was subjected to pyrolysis in a quartz tube furnace under Ar atmosphere, maintaining a temperature of 800 °C for 5 hours with a heating rate of 5 °C min^−1^. Similarly, for the synthesis of S-SiOC, with no pretreatment, 4 g of silicone oil with 0.8 g of 1-dodecanethiol were blended using a mortar and pestle. Subsequently, the mixture was transfered into an alumina boat and positioned at the inside of a tube furnace. The pyrolysis process was conducted under the same conditions used for SiOC, and the sample was retrieved after cooling down to room temperature.

### 2.3. Characterization

The weight loss of the materials within the temperature range of 25 to 1100 °C was measured using a thermogravimetric analysis (TGA) analyzer (Mettler Toledo). The heating rate was set at 5 °C min^−1^, and the measurements were conducted in an air atmosphere. To determine the Brunauer–Emmett–Teller (BET) surface areas and microstructural properties, a BELSORP-mini II instrument (MicrotracBEL Corp) was employed. Morphological analysis of the samples was carried out using a field-emission scanning electron microscopy (FE-SEM) with an energy dispersive X-ray spectrometer (EDS), specifically the Hitachi S-4800 model. For further characterization of morphology and structure, field-emission transmission electron microscopy (FE-TEM) was conducted using the JEOL JEM-2100F instrument, equipped with high-resolution transmission electron microscopy (HRTEM) and selected area electron diffraction (SAED) capabilities. X-ray diffraction (XRD) patterns were acquired utilizing a diffractometer, the Bruker D8-Advance 2020 model, with a wavelength (*λ*) of 1.5406 Å. Raman spectra were obtained employing a Raman spectrometer, specifically the LabRAM HR Evolution instrument, with a 532 nm laser source. Lastly, X-ray photoelectron spectroscopy (XPS) analysis was performed utilizing an X-ray photoelectron spectrometer from ThermoFisher Scientific, the NEXSA model.

### 2.4. Electrochemical measurement

The S-SiOC anode was prepared by creating a homogeneous mixture of active materials, Super-P as a conductive additive, and sodium carboxymethylcellulose (with a molecular weight of approximately 90 000, sourced from Sigma Aldrich) as a binder. The weight ratio of these components was 70 : 15 : 15. This mixture was thoroughly blended in deionized (DI) water using a Mini-Mill (PULVERISETTE 23 FRITSCH) for a duration of 30 minutes. Subsequently, the resulting mixture was spread onto a copper (Cu) foil using a doctor blade. The wet electrode was then dried at 60 °C in a vacuum oven for 4 hours. Following drying, the electrode was shaped into a disk with a diameter of 11 mm. The mass loading of the active material was maintained within the range of 0.7 to 2.2 mg cm^−2^. The assembly of CR2016 coin cells was carried out within an argon-filled glovebox (O_2_ and H_2_O < 1 ppm). These cells consisted of the S-SiOC material as the anode, a lithium (Li) metal disk serving as the counter electrode, and Celgard 2400 acting as the separator. The electrolyte was employed a 1.3 M solution of LiPF_6_ in a 3 : 7 (v/v) mixture of ethylene carbonate and diethyl carbonate, with the addition of 10% fluoroethylene carbonate obtained from PANAX. Electrochemical measurements were conducted using a WBCS3000S cycler from WonAtech. These measurements encompassed a voltage window spanning from 0.01 to 3 V (*versus* Li^+^/Li) and were conducted at room temperature. Cyclic voltammetry (CV) measurements were performed using the same WBCS3000S cycler over a voltage range of 0.01 to 3 V, employing scan rates ranging from 0.1 to 4.0 mV s^−1^. The galvanostatic intermittent titration technique (GITT) was employed to evaluate lithium-ion diffusivity during the second discharge–charge cycle, with the measurements also conducted using the WBCS3000S cycler. Finally, electrochemical impedance spectroscopy (EIS) analysis was carried out using a ZIVE SP1 instrument. The frequency range covered by this analysis spanned from 100 mHz to 100 kHz.

## 3. Results and discussion

**Fig. 1:**
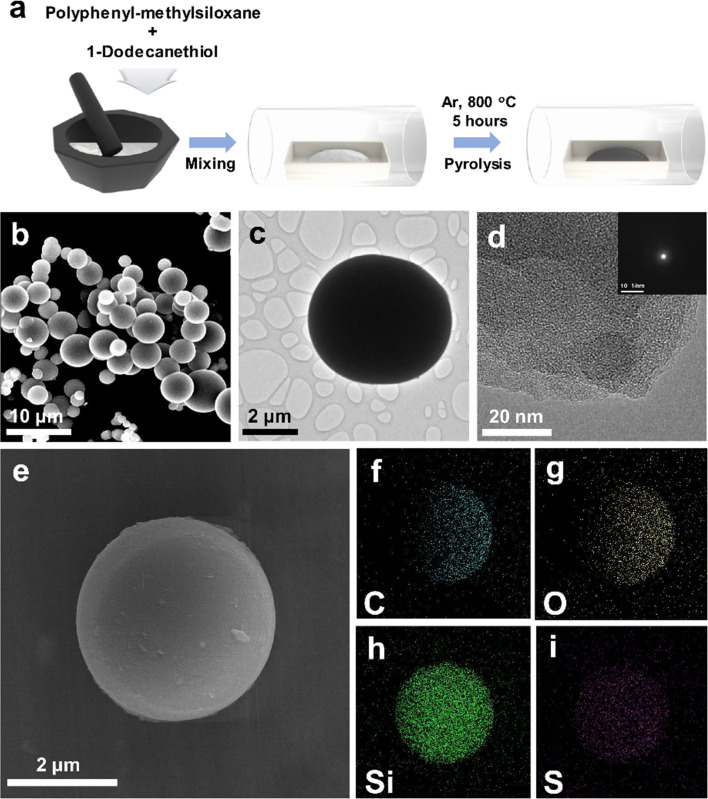
(a) Schematic illustration of preparing S-SiOC. (b) SEM image of S-SiOC. (c) TEM and (d) HRTEM images of S-SiOC (SAED pattern inset). (f) Carbon, (g) oxygen, (h) silicon, (i) sulfur elemental mappings of S-SiOC corresponding (e).

The schematic representation of the S-SiOC synthesis process is presented in Fig. 1a. Commercial silicone oil (polyphenyl-methylsiloxane) was utilized as the precursor, while 1-dodecanethiol served as the source of sulfur. Given the transparent nature of both silicone oil and 1-dodecanethiol, they were conveniently mixed without the need for supplementary solvents or surfactants.^35,36^ Subsequently, the mixture underwent pyrolysis at 800 °C under an Ar atmosphere. This process yielded black powders, indicative of the formation of carbon networks.^37^ The specific surface area of both SiOC and S-SiOC samples was assessed through N_2_ adsorption/desorption measurements. As illustrated in Fig. S1a,† the SiOC and S-SiOC materials were characterized by type II and type IV isotherms, respectively. BET analysis of SiOC and S-SiOC revealed nonporous attributes, featuring low specific surface areas of 0.257 m^2^ g^−1^ and 2.033 m^2^ g^−1^, respectively. The pore size distribution of SiOC and S-SiOC was examined using the BJH plot, as displayed in Fig. S1b.† The total pore volumes are 0.00141 cm^3^ g^−1^ and 0.00371 cm^3^ g^−1^, respectively.

As shown in Fig. 1b and S2a,† morphologies and particle dimensions are illustrated in the SEM images. Both samples display particles primarily in a spherical form, featuring micron-sized distributions. In particular, the introduction of sulfur through doping manifests no prominent differences in particle morphology between two samples, as evident in the SEM images. The particle sizes of SiOC and S-SiOC can be determined *via* analysis of SEM images. The particle sizes of SiOC and S-SiOC range from 1.4 to 5.4 μm and 2.9 to 9.6 μm, respectively, demonstrating a diverse distribution of particles in terms of size. For a more comprehensive exploration of morphology and structure, TEM images of SiOC and S-SiOC are presented in Fig. 1c and S2b.† In both cases, bulk particles exhibiting spherical configurations were consistent with observations from SEM images. HRTEM images and SAED analyses of SiOC and S-SiOC are displayed in Fig. 1d and S2c,† respectively. These images showed that both samples possess a fully amorphous structure devoid of crystallinity. This outcome shows that the pyrolysis temperature did not suffice to induce crystalline formation. To further verify elemental composition, EDS elemental mapping was employed, and results are shown in Fig. 1f–i and S2e–h.† This analysis was confirmed the uniform distribution of Si, O, and C elements, while S was not detected within SiOC. In contrast, S-SiOC exhibited homogeneous distributions of Si, O, C, and S elements. EDS spectra of both samples are presented in Fig. S3.† In pure SiOC, distinct peaks were observed for Si, O, and C, with no signal indicative of S. Conversely, in sulfur-doped SiOC, all the expected peaks were observed, including a visible S spectrum (∼1.83% in atomic ratio). Notably, EDS elemental mapping images provided compelling evidence that sulfur atoms have been effectively doped into the silicon oxycarbide structure.

**Fig. 2:**
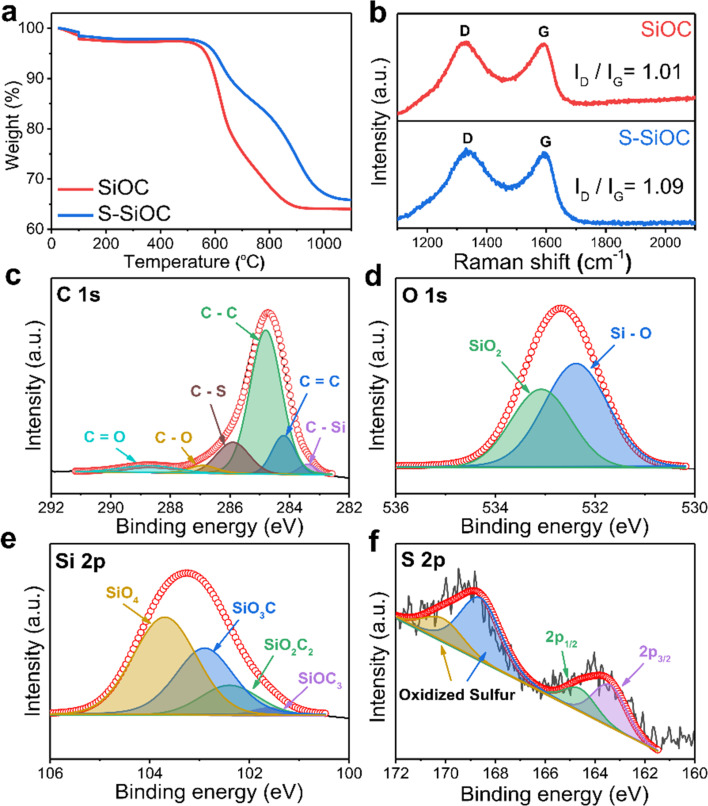
(a) TGA curves of SiOC and S-SiOC at a heating rate of 5 °C min^−1^ under the air atmosphere. (b) Raman spectra of SiOC and S-SiOC. High resolution XPS spectra of (c) C 1s, (d) O 1s, (e) Si 2p, (f) S 2p in S-SiOC.

Fig. 2a illustrates TGA curves of SiOC and S-SiOC across the temperature range of 25 to 1100 °C under an air atmosphere. For both samples, the initial weight loss occurring below 200 °C was attributed to water evaporation. Meanwhile, the primary weight reduction observed above 500 °C was attributed to the oxidation of free carbon in the presence of air.^37^ Notably, the reduced weight percentages for SiOC and S-SiOC are 33.8% and 32.7%, respectively, indicating the establishment of a carbon network with minimal variation in carbon content between the two samples. Meanwhile, the content of the free carbon phase in SiOC plays a vital role in influencing electrochemical activity. This finding suggests that the difference in electrochemical activity between the two samples is not influenced by the free carbon phase.

Fig. S4† presents the X-ray diffraction (XRD) patterns of the as-prepared SiOC and S-SiOC samples. In SiOC, the broad diffraction peak at 2*θ* = 22.8° corresponds to the (002) reflection and signifies the presence of locally graphitized carbon structures within the amorphous carbon phase. Upon sulfur doping, the intensity of the (002) peak in S-SiOC decreased and shifted to a lower value of 21.4°. The calculated *d*_002_ values (representing the interlayer spacing) for SiOC and S-SiOC are 0.390 nm and 0.415 nm, respectively. This expanded interlayer spacing is beneficial for lithium-ion storage and diffusion. Sulfur's larger ionic radius in comparison to carbon contributes to the extension of the carbon interlayer spacing.^38–41^ Notably, the XRD patterns for S-SiOC do not exhibit peaks indicative of pristine sulfur, confirming that sulfur does not exist in an elemental state.^29^ The broadness and intensity of the XRD patterns signify that SiOC maintains its amorphous structure, consistent with previous reports.^36,42–44^ Similar patterns persist in S-SiOC, indicating that doped sulfur atoms do not alter the amorphous structure of SiOC. Furthermore, the absence of SiC crystallization aligns with the SAED patterns, as the pyrolysis temperature is insufficient for SiC carbothermal reduction.^37,45^ The absence of SiC is advantageous for achieving enhanced reversible capacity due to its inertness towards lithium ions.^46^ To provide more qualitative information, the domain sizes of the samples were analyzed utilizing the Scherrer equation.^47–49^ The domain sizes of SiOC and S-SiOC, as determined from the XRD patterns, are measured to be 2.12 nm and 1.83 nm, respectively.

The Raman spectra of both SiOC and S-SiOC are shown in Fig. 2b. Two major peaks at 1330 cm^−1^ and 1592 cm^−1^ were observed, which are generally observed in carbonaceous materials.^25,44,45,50,51^ The former peak is related to the disordered carbon (D band) and the latter is associated with the graphitized carbon (G band), respectively. The intensity ratio of the D and G band (*I*_D_/*I*_G_), which generally refers to the ordering degree of carbon, increased from 1.01 to 1.09 after sulfur doping. This result reflects the increase of defects and degree of disorder in S-SiOC material due to doped-sulfur atoms, which can supply more diffusion channels and active sites for Li^+^ insertion.^42^ It is consistent well with the XRD result.

The surface chemical composition of S-SiOC was assessed *via* X-ray photoelectron spectroscopy (XPS). Fig. 2c–f presents high-resolution spectra of C 1s, O 1s, Si 2p, and S 2p in S-SiOC along with the corresponding fitted results. In Fig. 2c, the C 1s peak was deconvoluted into six distinct peaks: C–Si (283.4 eV), C=C (284.2 eV), C–C (284.8 eV), C–S (285.9 eV), C–O (286.9 eV), and C=O (288.7 eV). The O 1s peak yielded two clear peaks: Si–O (532.38 eV) and SiO_2_ (533.08 eV) in Fig. 2d. The C 1s spectrum revealed an elevated peak intensity for C–C/C=C in S-SiOC, along with a distinctly observable C–S bonding peak, as compared to SiOC shown in Fig. S5a.† This observation suggests that doped sulfur atoms influenced the distribution of C–Si and C–C/C=C bonds, thereby modifying the structure of both the Si–O–C glass phase and the free carbon phase. Similarly, the O 1s spectrum in Fig. 2d shows comparable Si–O/SiO_2_ bonding peaks post-sulfur doping, concerning bare SiOC shown in Fig. S5b.† The Si 2p peak in Fig. 2e can be resolved into four peaks: SiOC_3_ (101.6 eV), SiO_2_C_2_ (102.4 eV), SiO_3_C (102.9 eV), and SiO_4_ (103.7 eV), consistent with previous findings.^37,52,53^ In particular, Fig. S5c† shows that the deconvoluted peaks display minimal changes following sulfur doping in comparison to bare SiOC. This suggests that structural adjustments between the two phases occurred without the formation of covalent bonds between silicon and sulfur atoms. However, a distinct contrast emerges in the S 2p spectrum, highlighting the pronounced differences between the two samples. The high-resolution S 2p spectrum for S-SiOC is characterized by four peaks at 163.4 eV, 164.6 eV, 168.6 eV, and 170.1 eV in Fig. 2f. The initial two peaks, separated by 1.2 eV, are attributed to S 2p_3/2_ and S 2p_1/2_, corresponding to C–S–C bonding with a heterocyclic structure.^23,28,29,41^ The latter two peaks correspond to oxidized sulfur groups.^54^ This observation indicates the successful establishment of covalent bonds between sulfur and carbon atoms.^23,51^ Conversely, in Fig. S5d,† the S 2p spectrum for SiOC does not display any obvious peak.

XPS analysis was performed on the interfaces of SiOC and S-SiOC anodes following the initial cycle.^55,56^ Firstly, Fig. S6a† shows the F 1s peak of SiOC and S-SiOC. In the F 1s spectrum of SiOC, two peaks appear at 685.9 and 687.6 eV, corresponding to LiF and CF_3_, respectively. For S-SiOC, the F 1s peaks are located at 684.7 eV and 687.2 eV for LiF and CF_3_, respectively. Here, LiF indicates the formation of the anode's SEI, while CF_3_ refers to byproducts resulting from the decomposition of the electrolyte.^57^ Comparing the LiF to CF_3_ ratio between SiOC and S-SiOC, it is evident that S-SiOC exhibits a higher LiF ratio of 87.7% compared to SiOC's 77.4%. Therefore, it can be concluded that sulfur doping positively influences SEI formation.^58^ Fig. S6b† displays the O 1s peaks for SiOC and S-SiOC. The O 1s spectrum for SiOC reveals peaks at 528.5 and 531.9 eV, which correspond to Li_2_O and LiCO_*x*_, respectively. Similar peaks at 528.5 (Li_2_O) and 531.9 eV (LiCO_*x*_) are also observed in S-SiOC. Given that Li_2_O is one of the components of the SEI layer, the relatively larger Li_2_O peak in S-SiOC suggests that it has been influenced by sulfur doping.^58^ Fig. S6c† displays the C 1s peak of SiOC and S-SiOC. While it resembles the C 1s spectrum of original SiOC, we observed the emergence of C–H and R–O–C peaks at 283.2 eV and 287 eV, respectively, after cycling.^58–60^ These peaks represent the bonds of carbon and organic substances formed alongside the SEI layer post-cycling. In Li-ion batteries, the combination of carbon and organic materials adversely affects the SEI layer, unlike inorganic bonds.^61^ The increased peaks of carbon-organic bonds in SiOC compared to S-SiOC suggests that the SEI stability of SiOC may be some what lower than that of S-SiOC.

**Fig. 3:**
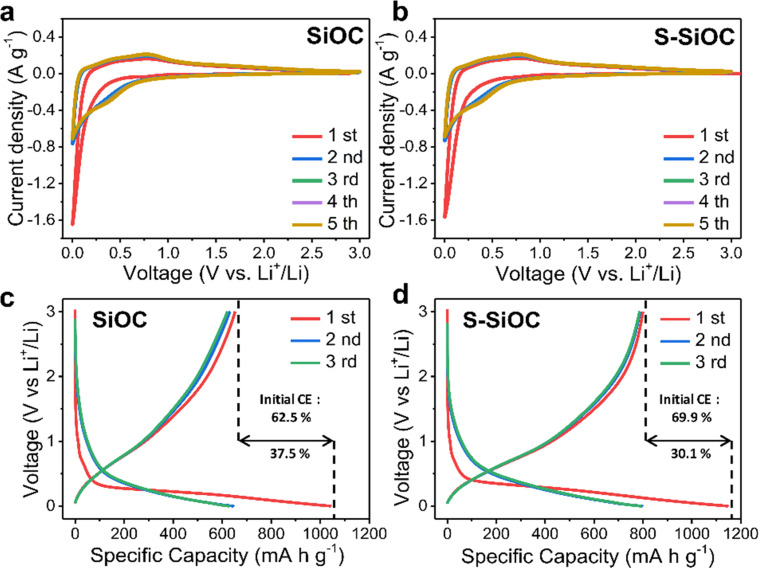
Cyclic voltammetry for the initial 5 cycles of (a) SiOC and (b) S-SiOC, at a scan rate of 0.1 mV s^−1^. Galvanostatic profiles of (c) SiOC and (d) S-SiOC for the initial 3 cycles. The current density is 0.1 A g^−1^.

CV was conducted at a scan rate of 0.1 mV s^−1^ to provide insights into the electrochemical reactions. In Fig. 3b, The CV curves for the S-SiOC electrode exhibited a broad anodic peak and a distinct cathodic peak, both occurring at around 0.01 V (*vs.* Li^+^/Li). These features was attributed to the insertion or extraction of Li^+^ within the amorphous SiOC structure.^44,62,63^ Likewise, as shown in Fig. 3a, the SiOC electrode displays comparable CV curves, characterized by a broad anodic peak and a sharp cathodic peak. The faint cathodic peak observed at 0.7–0.8 V indicates the formation of a solid electrolyte interface (SEI) layer.^64^ Moreover, overlapping curves of both samples from the second to fifth cycles indicate a highly reversible lithiation/delithiation reaction. Especially, response current was elevated in the S-SiOC electrode, a trend attributable to enhanced electrical conductivity resulting from the doping process.

Galvanostatic charging and discharging curves for both SiOC and S-SiOC are demonstrated in Fig. 3c and d during the initial three cycles at a current rate of 0.1 A g^−1^ within the voltage range of 0.01 to 3 V. In the initial cycle, the discharge curve displays a plateau at 0.7–0.8 V, aligning with the CV curves. The initial cycle discharge capacities for SiOC and S-SiOC at 0.1 A g^−1^ was recorded as 1042 mA h g^−1^ and 1146 mA h g^−1^, respectively. SiOC demonstrates a reversible specific capacity of 652 mA h g^−1^, accompanied by an initial coulombic efficiency (ICE) of 62.5%. On the other hand, S-SiOC exhibited an elevated reversible capacity of 800 mA h g^−1^ and an improved ICE of 69.9%. The observed low ICE could stem from irreversible side reactions associated with the binding of lithium to oxygen-containing functional groups (*e.g.*, C=O, C–OH) or active carbon sites, which subsequently leads to the formation of solid electrolyte interface (SEI) layers on the anode surface.^44,46^ These functional groups, contributing to the non-reversible storage of lithium ions, could be eliminated through sulfur doping reactions, either by SO_*x*_ oxidation or S substitution, resulting in an enhancement of coulombic efficiency.^65^ Furthermore, considering the similarity in specific surface area between the two samples based on BET results, the distinction in ICE could likely be attributed to the sulfur doping reaction. The second cycle discharge capacities for SiOC and S-SiOC was measured at 645 mA h g^−1^ and 797 mA h g^−1^, respectively. Correspondingly, ensuing reversible specific capacities and coulombic efficiencies for these cycles was measured as 630 mA h g^−1^ with 97.7% and 788 mA h g^−1^ with 98.9%. Importantly, as cycles progress, the coulombic efficiencies demonstrate an inclination to increase.

**Fig. 4:**
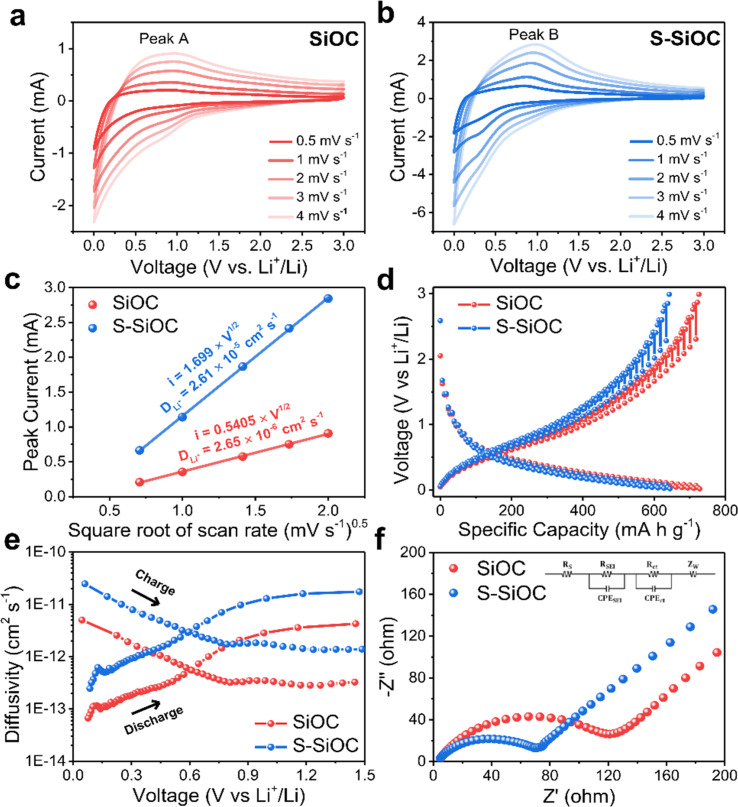
Cyclic voltammetry curves of (a) SiOC and (b) S-SiOC with different scan rates from 0.5 mV s^−1^ to 4 mV s^−1^. (c) Linear fitting of the current at each anodic peak to the corresponding scan rate. (d) GITT data of SiOC and S-SiOC during the second discharge–charge cycle, current pulse of 0.1 A g^−1^. (e) Diffusivity plot of SiOC and S-SiOC during charge and discharge, and which evaluated by GITT. (f) Nyquist plots of SiOC and S-SiOC electrodes after 100 cycles at 1 A g^−1^ and equivalent circuit model (inset). (*R*_s_: electrolyte resistance, CPE_SEI_: SEI layer capacitance, *R*_SEI_: SEI layer resistance, CPE_ct_: double layer capacitance, *R*_ct_: charge transfer resistance, and *Z*_w_: Warburg diffusion element.)

To assess the impact of doping on electrochemical properties, CV curves were conducted at different scan rates for both SiOC and S-SiOC, and are shown in Fig. 4a and b. The scan rate ranged from 0.5 mV s^−1^ to 4 mV s^−1^. Especially, as the scan rate escalates, there is a tendency for the peak current to increase in both samples. This phenomenon is likely linked to the activation of electrochemical reactions.^44^ For a deeper understanding of the role of sulfur doping in electrochemical performance, a linear correlation between the anodic peak current and the square root of the scan rate was established and displayed in Fig. 4c. Specifically, Fig. 4a and b are depicted Peak A of SiOC, and Peak B of S-SiOC, respectively. The diffusion coefficient of lithium-ion, *D*_Li^+^_, was subsequently calculated from the CV curves with varying scan rates using the Randles–Sevcik equation (eqn (1)).

1 *I*_p_ = 2.69 × 10^5^*n*^1.5^*AD*_Li_^0.5^*v*^0.5^*C*_Li_

In this equation, *I*_p_ signifies the peak current, *A* represents the anode area, *n* corresponds to the number of electrons in the relevant reaction, *v* denotes the scan rate, and *C*_Li_ symbolizes the concentration of lithium-ions within the electrolyte.^66,67^ Upon plotting and employing the equation, the slope was determined to be 2.65 × 10^−6^ for SiOC and 2.61 × 10^−5^ for S-SiOC. Particularly, the calculated value for S-SiOC is over tenfold greater than that of SiOC. This difference indicates that sulfur doping significantly enhances the electrochemical kinetics of Li^+^.^68,69^

For a more in-depth exploration of lithium-ion diffusivity, GITT was executed on both SiOC and S-SiOC throughout the second discharge–charge cycle. The GITT curve for both samples within the voltage range of 0.01 to 3.0 V is depicted in Fig. 4d. This technique involved applying a current pulse of 0.1 A g^−1^ for a pulse time of 10 minutes, followed by a relaxation time of 60 minutes. To determine the diffusivity of lithium-ion (*D*_Li^+^_), the calculation employed eqn (2), initially formulated by Weppner and Huggins. This method aids in assessing the rate at which lithium ions diffuse within the material.2
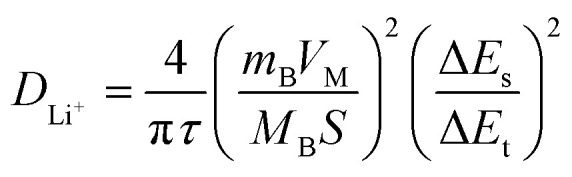
In this equation, *τ* represents the pulse time, *m*_B_ signifies the mass of active materials, *V*_M_ denotes the molar volume of the active materials, *M*_B_ represents the molar mass of the active materials, *S* symbolizes the interface surface area of the electrode, ΔE_s_ refers to the voltage change during a single-step experiment, and Δ*E*_t_ represents the total change in cell voltage during a constant current pulse.^70^ However, complexity of SiOC samples makes the determination of *V*_M_ and *M*_B_ challenging due to the composite nature of the material, involving varying amounts of SiO_*x*_C_*y*_ glass phase and the C_free_ phase with differing *x* and *y* values. Consequently, this equation can be simplified to eqn (3) for SiOC samples.3
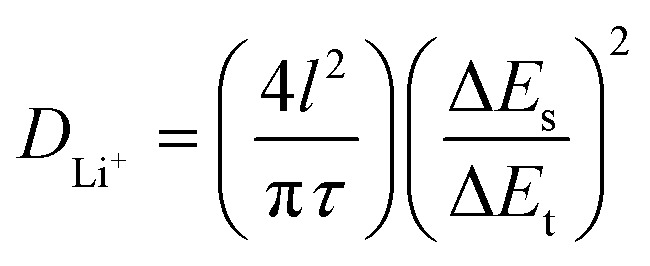
*l* represents the characteristic diffusion length, which can also be interpreted as the average particle radius, measuring 1.137 μm.^70,71^ The voltage profile of the second cycle and the corresponding calculated *D*_Li^+^_ values for both electrodes are presented in Fig. 4d, e, and S6.† Notably, the diffusion coefficient of the S-SiOC electrode consistently surpasses that of SiOC across different potentials during both the charging and discharging processes. This alignment with the CV results strongly suggests that the heightened lithium-ion diffusivity can be attributed to sulfur doping. Sulfur doping is believed to substantially reduce the energy barrier for diffusion during both lithiation and delithiation processes.^72^

Subsequently, electrochemical impedance spectroscopy (EIS) was conducted to gain insights into the enhanced electrochemical kinetics of both SiOC and S-SiOC electrodes after 100 cycles. The Nyquist plots of the electrodes, accompanied by their equivalent circuit, are displayed in Fig. 4f, while the EIS fitting outcomes are provided in Table S1.† These plots split into distinct components, including electrolyte resistance (*R*_s_), resistance associated with the solid–electrolyte interface (*R*_SEI_), and charge transfer resistance (*R*_ct_). Furthermore, the straight line observed in the low-frequency region, known as the Warburg line, corresponds to lithium-ion diffusion occurring between the electrolyte and the electrode interface.^73^ After 100 cycles, it's noteworthy that the *R*_s_ and *R*_SEI_ values for the S-SiOC electrode are closely aligned with those of the SiOC electrode, measuring 4.57 Ω and 12.2 Ω, respectively, and 4.69 Ω and 12.8 Ω, respectively. However, the *R*_ct_ value of the S-SiOC electrode, standing at 43.0 Ω, is approximately half the magnitude of the SiOC electrode's *R*_ct_ value of 87.2 Ω. These outcomes strongly indicate that sulfur doping contributes to an enhancement in electrical conductivity and an increase in ionic diffusion within the electrode. This phenomenon can be attributed to the sulfur-induced expansion of the carbon interlayer spacing, coupled with its potential to lower the energy barrier for lithium-ion diffusion. Additionally, sulfur can provide supplementary pathways for electron transfer, facilitated by the presence of C–S–C bonding, as highlighted in the XRD results.^32,38,72^

**Fig. 5:**
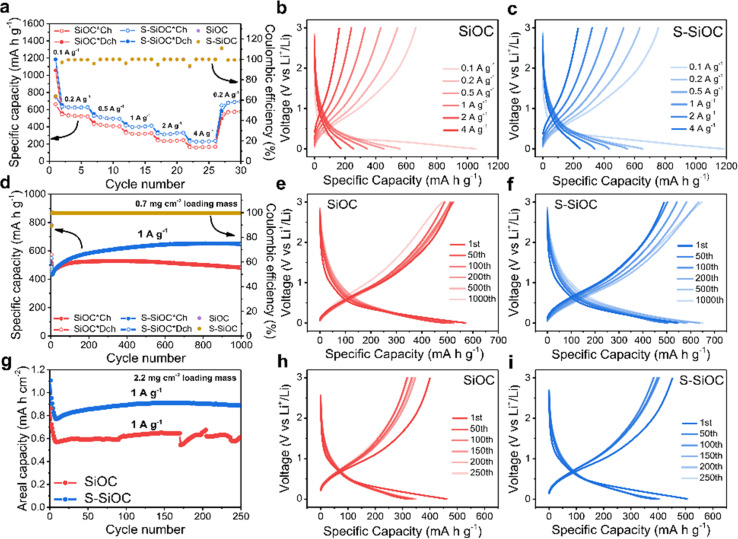
(a) Rate performance of SiOC and S-SiOC at different current densities. Corresponding voltage profiles of (b) SiOC and (c) S-SiOC at various current densities. (d) Cycling performance and coulombic efficiency of SiOC and S-SiOC at 1 A g^−1^ for 1000 cycles (0.7 mg cm^−2^ of loading mass) and (e and f) corresponding voltage profiles at current density of 1 A g^−1^. (g) Discharge areal capacity plot of SiOC and S-SiOC at 1 A g^−1^ for 250 cycles (2.2 mg cm^−2^ of loading mass) and (h and i) corresponding voltage profiles at current density of 1 A g^−1^.

The rate performance of both SiOC and S-SiOC electrodes was evaluated across different current densities, as depicted in Fig. 5a–c. Specifically, SiOC electrodes delivered reversible specific capacities of 529, 416, 322, 238, and 164 mA h g^−1^ at current densities of 0.2, 0.5, 1, 2, and 4 A g^−1^, respectively. Through the heightened electrical conductivity afforded by sulfur doping, the S-SiOC electrodes exhibited significantly elevated capacities of 626, 505, 406, 312, and 231 mA h g^−1^ at the same respective current densities. Furthermore, as the current density increased, it became evident from Fig. 5b and c that the degree of overpotential was more pronounced in bare SiOC compared to S-SiOC. Interestingly, when the current density was lowered from 4 A g^−1^ to 0.2 A g^−1^, a notable increase in reversible specific capacities was observed. Specifically, larger reversible specific capacities of 570 mA h g^−1^ and 680 mA h g^−1^ were recorded for SiOC and S-SiOC electrodes, respectively, upon the current density's reduction. These outcomes could likely be attributed to the activation of the electrodes during cycling, which contributes to the increased capacities.

The cycling performances of the electrodes were assessed at a current density of 1 A g^−1^, as illustrated in Fig. 5d–f. Both the SiOC and S-SiOC electrodes display activation processes and impressive cycling stability, attributable to the inherent structural stability within the SiOC-based electrode.^7^ This activation process could potentially be attributed to the micro-sized nature of the produced SiOC particles, as well as their dense surfaces.^63^ Remarkably high coulombic efficiencies of 99.6% were consistently observed for both types of electrodes throughout the 1000 consecutive cycles. However, S-SiOC electrode exhibited a remarkable increase in reversible capacity throughout cycling, indicating ongoing activation. As results of Fig. 5e and f, this dynamic activation process contributed to the S-SiOC electrode achieving a higher specific capacity of 650 mA h g^−1^ with no capacity fading, in contrast to the SiOC electrode's specific capacity of 480 mA h g^−1^ and capacity retention of 83.9% after 1000 cycles. Furthermore, as shown in Fig. S8,† S-SiOC electrode showed exceptional capacity retention, with an impressive 89.2% retention after 2000 cycles at 1 A g^−1^. To evaluate the high loading potential of the S-SiOC electrode, we fabricated electrodes with a mass loading of 2.2 mg cm^−2^. As depicted in Fig. 5g, the S-SiOC electrode attained a discharge areal capacity surpassing 0.88 mA h cm^−2^ during 250 cycles at 1 A g^−1^. In contrast, SiOC electrode demonstrated relatively lower areal capacity under the same conditions. Moreover, Fig. 5h and i indicates that the cycle stability of S-SiOC electrode was notably superior, with maintaining a capacity retention of 80.6% after 250 cycles, while bare SiOC showed 69.5% remaining capacity. This exhibits the enhanced electrical performance of the S-SiOC electrode, which can be largely attributed to the effect of sulfur doping. While many SiOC-based electrodes contend with challenges related to electrically insulating characteristics and poor ionic transport, the S-SiOC electrodes exhibit improved electrical conductivity, thereby leading to exceptional cycle and rate performance. Our synthesis method and electrochemical performance data on S-SiOC as the anode are compared to the data found in the literature, as shown in Table S2.† To provide qualitative information on the durability of the S-SiOC electrode, surface analysis was conducted following cycling. The SEM images of the S-SiOC electrode before and after cycling are shown in Fig. S9a and b.† Both images display the morphologies of the S-SiOC electrode fabricated using a CMC binder and carbon. After cycling test, it was observed that there were no substantial agglomerations or cracks compared to the pre-cycling state, and the morphology displayed similarities. The TEM analysis findings of the S-SiOC electrode post cycling indicated that the spherical morphology of the S-SiOC electrode was sustained, as depicted in Fig. S9c.† Consequently, the SEM and TEM analyses conducted post cycling indicated that the morphology of the S-SiOC remained predominantly unchanged, thereby highlighting the stability of S-SiOC.^74,75^

## 4. Conclusion

In summary, successful synthesis of sulfur-doped silicon oxycarbide (S-SiOC) through a facile pyrolysis process of commercial silicone oil marks a significant advancement in the field. This study has demonstrated the exceptional capabilities of S-SiOC as an anode material for lithium-ion batteries (LIBs). The electrode exhibits noteworthy attributes including a substantial specific capacity, remarkable capacity retention, and the ability to accommodate a high mass loading of active materials. These outstanding electrochemical performances are directly linked to the enhanced conductivity of S-SiOC, a consequence of the sulfur doping strategy employed. The experimental findings offer compelling evidence that the introduction of sulfur atoms enhances the material's capacity to store Li^+^ ions, resulting in improved Li^+^ diffusion rates and overall rate capability. Additionally, the formation of covalent bonds between carbon and sulfur atoms contributes to the heightened electrical conductivity of the material. As a result, the S-SiOC electrode emerges as a highly promising candidate for anode applications in lithium-ion batteries. The demonstrated electrochemical performance of S-SiOC, coupled with its facile synthesis process, positions it as a promising potential for advancing the performance of energy storage systems and driving the evolution of battery technologies.

## Data availability

The data supporting this article have been included as part of the ESI.†

## Conflicts of interest

There are no conflicts to declare.

## Supplementary Material

RA-014-D4RA04608K-s001
