# Supplementary material for: Sulfur-doped silicon oxycarbide by facile pyrolysis process as an outstanding stable performance lithium-ion battery anode
Source: RSC Adv. 2024 Sep 20;14(41):29999–30010. doi: 10.1039/d4ra04608k (PMC11413737; doi:10.1039/d4ra04608k)
Supplement: RA-014-D4RA04608K-s001 [file RA-014-D4RA04608K-s001.pdf]

Supplementary Information (SI) for RSC Advances.
This journal is © The Royal Society of Chemistry 2024

Supplementary information

Sulfur-doped Silicon Oxycarbide by Facile Pyrolysis Process as an Outstanding Stable Performance Lithium-Ion Battery Anode

Jungjin Park [a,1], Won Young An [a,1], Keunho Lee [a], Seungman Park [a], Minjun Bae [a], Seon Jae Hwang [a], Hwichan Hong [a], Yonghwan Kim [a], Taehyun Yoo [a], Dohyeong Kim [a], Jong Min Kim [c,], and Yuanzhe Piao [a,b,*]*

[a] Graduate School of Convergence Science and Technology, Seoul National University, 145 Gwanggyo-ro, Yeongtong-gu, Suwon-Si, Gyeonggi-do, 16229, Republic of Korea

[b] Advanced Institutes of Convergence Technology, 145 Gwanggyo-ro, Yeongtong-gu, Suwon-si, Gyeonggi-do, 16229, Republic of Korea

[c] Samsung Electro-Mechanics, 150, Maeyeong-ro, Yeongtong-gu, Suwon-si, Gyeonggi-do, 16674, Republic of Korea

*Corresponding Authors

E-mail: parkat9@snu.ac.kr (Y. Piao)

E-mail: vitamin66@snu.ac.kr (J. M. Kim)

Fig. S1 N_2 adsorption/desorption isotherms of (a) SiOC and (b) S-SiOC. (BET surface area of SiOC, S-SiOC is 0.257 m^2 g^{-1}, 2.033 m^2 g^{-1} respectively.) (c) BJH pore size distribution for the SiOC and S-SiOC.

Fig. S2 Morphology and microstructure of SiOC.

Fig. S3 Quantitative analysis spectra and atomic percentage of (a) SiOC and (b) S-SiOC corresponding from EDS analysis (inset).

Fig. S4 XRD patterns of SiOC and S-SiOC.

Fig. S5 High resolution XPS spectra of (a) C 1s, (b) O 1s, (c) Si 2p and (d) S 2p in SiOC.

Fig. S6 XPS spectra of (a) F 1s, (b) O 1s, and (c) C 1s in SiOC and S-SiOC anode after initial cycle.

Fig. S7 Voltage profile of (a) SiOC and (b) S-SiOC during GITT analysis. (c) Demonstration of a single titration with ΔE_t, ΔE_sparameters. (d) Relationship between the voltage and square root of the pulse time at a single current pulse during GITT analysis of both electrodes.

Fig. S8 Cycle performance and coulombic efficiency of S-SiOC electrode at 1 A g^{-1} for 2000 cycles (0.7 mg cm^{-2} of loading mass).

Fig. S9 SEM images of S-SiOC (a) before and (b) after cycling test. (c) TEM image of S-SiOC after cycling test.

Table S1 EIS fitting results of SiOC and S-SiOC electrodes.

Table S2 Comparison of electrochemical performances between the S-SiOC electrode and other reported SiOC-based electrodes.

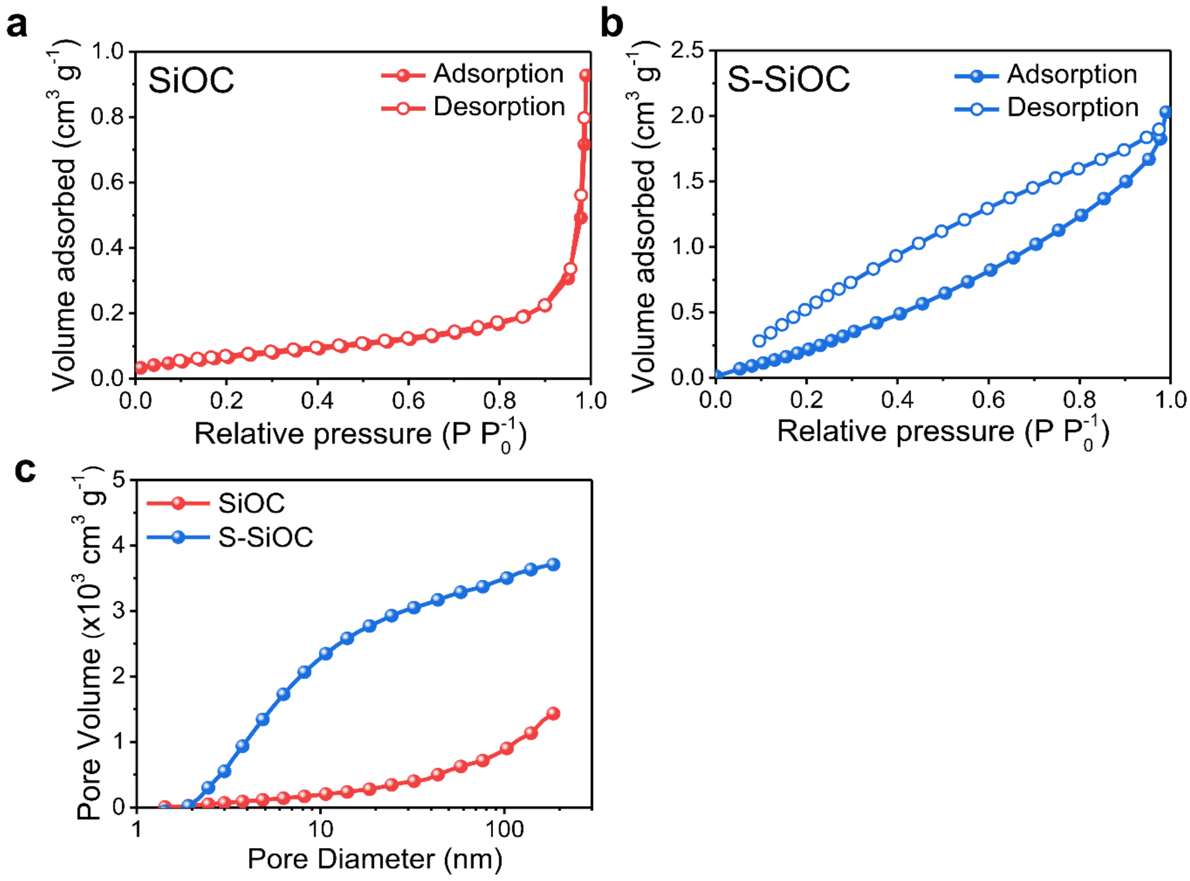

Fig. S1. N_2 adsorption/desorption isotherms of (a) SiOC and (b) S-SiOC. (BET surface area of SiOC, S-SiOC is 0.257 m^2 g^{-1}, 2.033 m^2 g^{-1} respectively.) (c) BJH pore size distribution for the SiOC and S-SiOC.

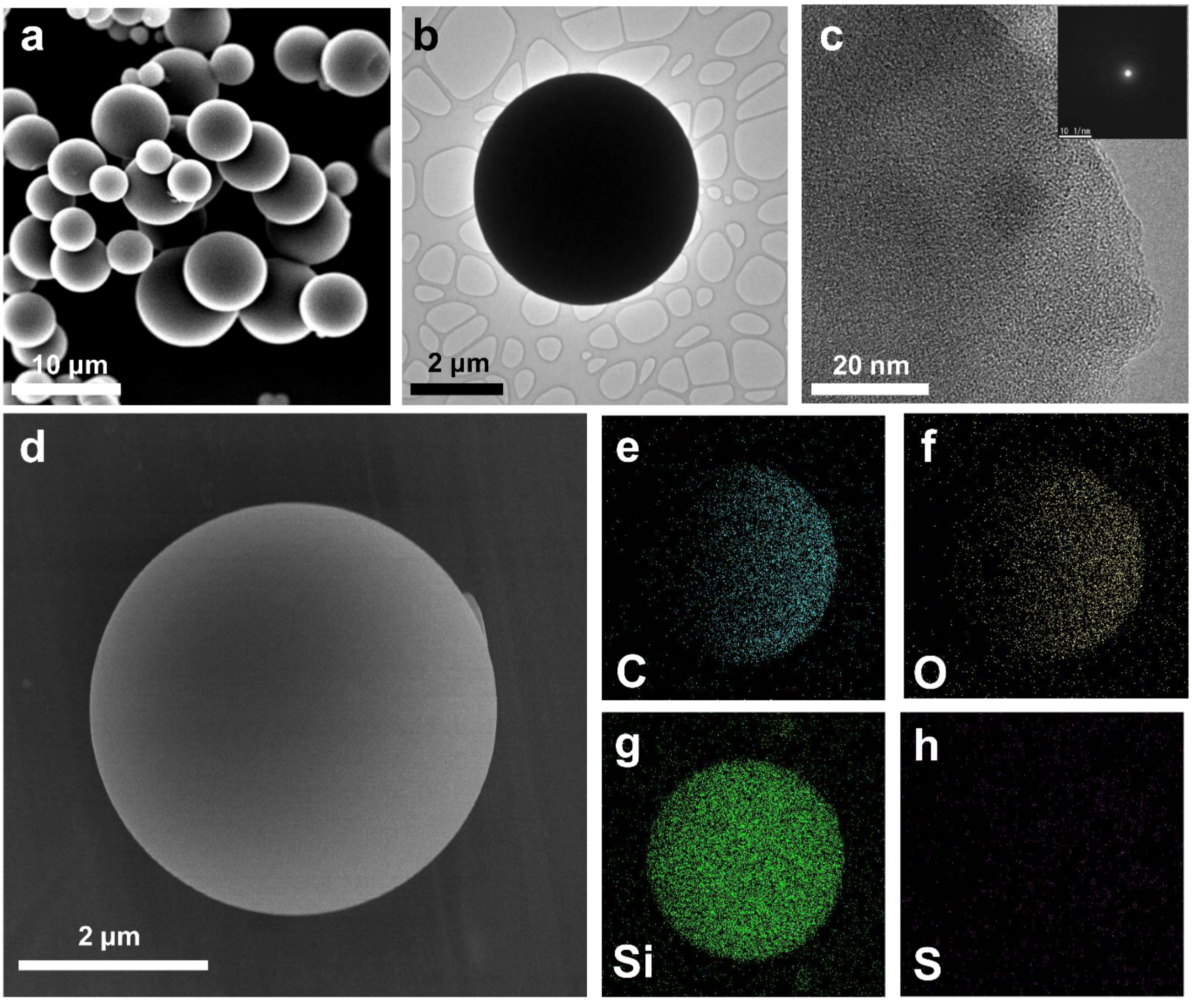

Fig. S2. Morphology and microstructure of SiOC. (a) SEM image of SiOC; (b) TEM and (c) HRTEM images of SiOC (SAED pattern inset); (e) Carbon, (f) oxygen, (g) silicon, (h) sulfur elemental mappings of SiOC corresponding (d).

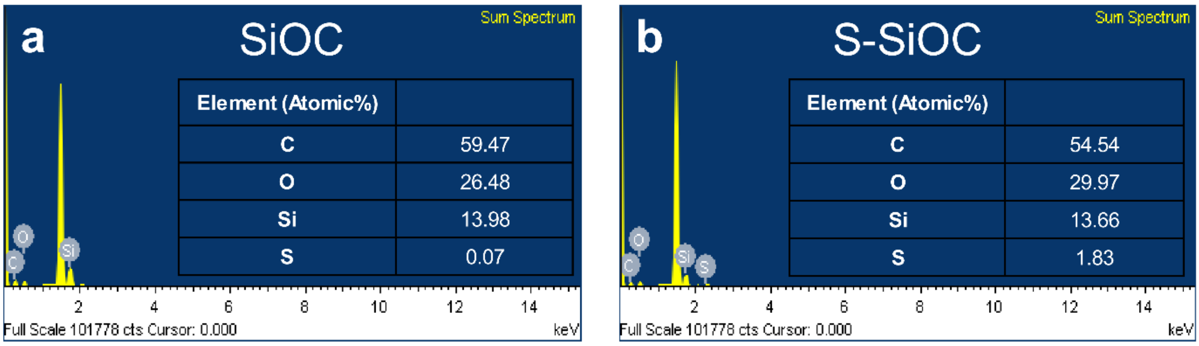

Fig. S3. Quantitative analysis spectra and atomic percentage of (a) SiOC and (b) S-SiOC corresponding from EDS analysis (inset).

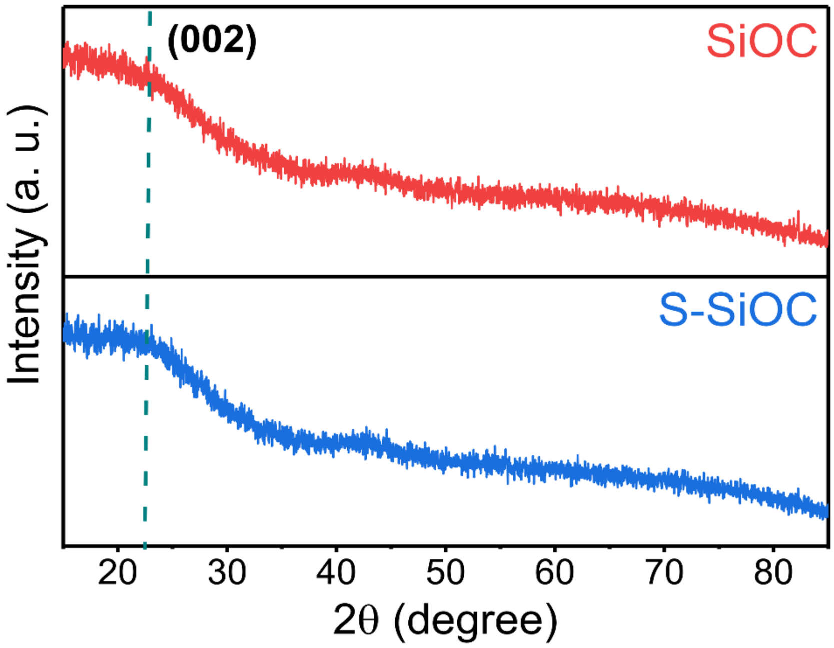

Fig. S4. XRD patterns of SiOC and S-SiOC.

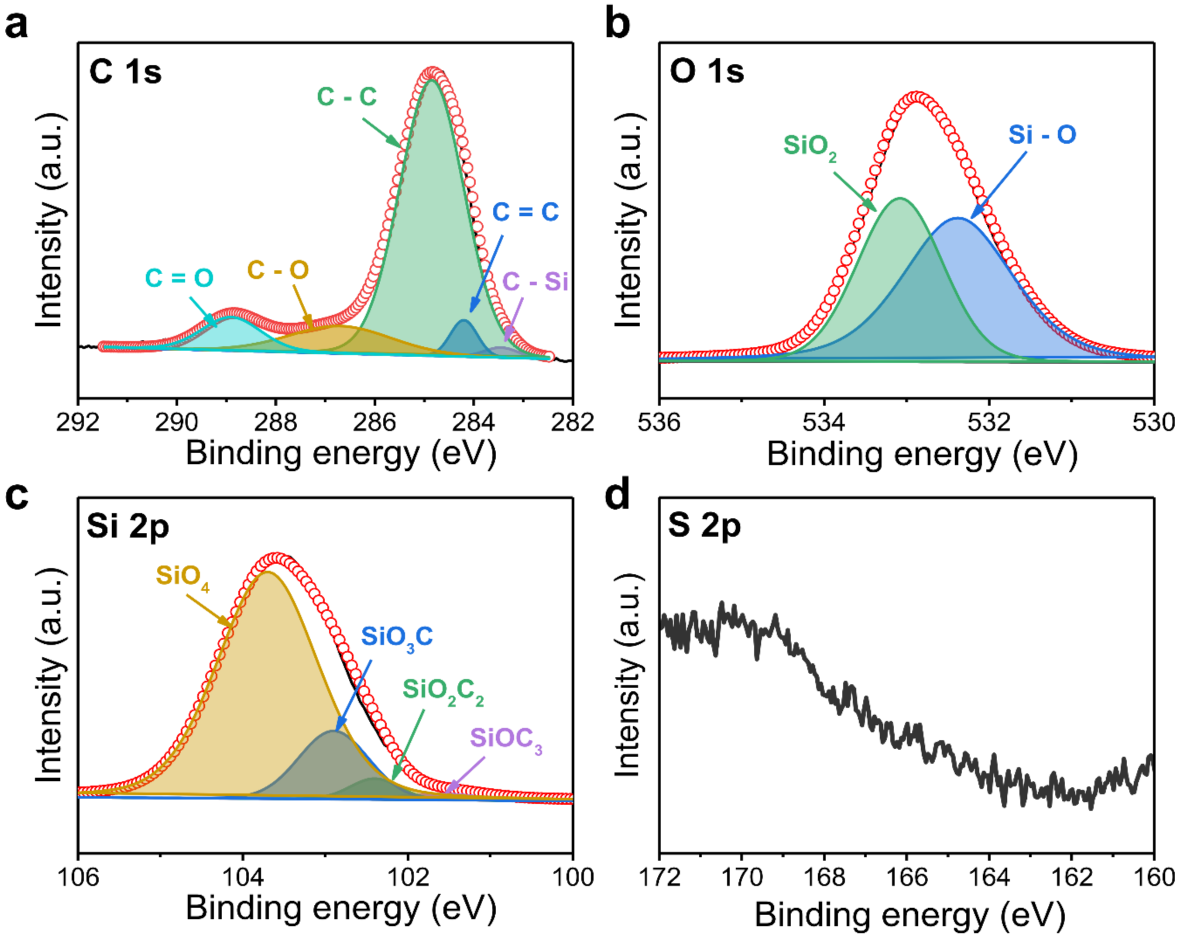

Fig. S5. High resolution XPS spectra of (a) C 1s, (b) O 1s, (c) Si 2p and (d) S 2p in SiOC.

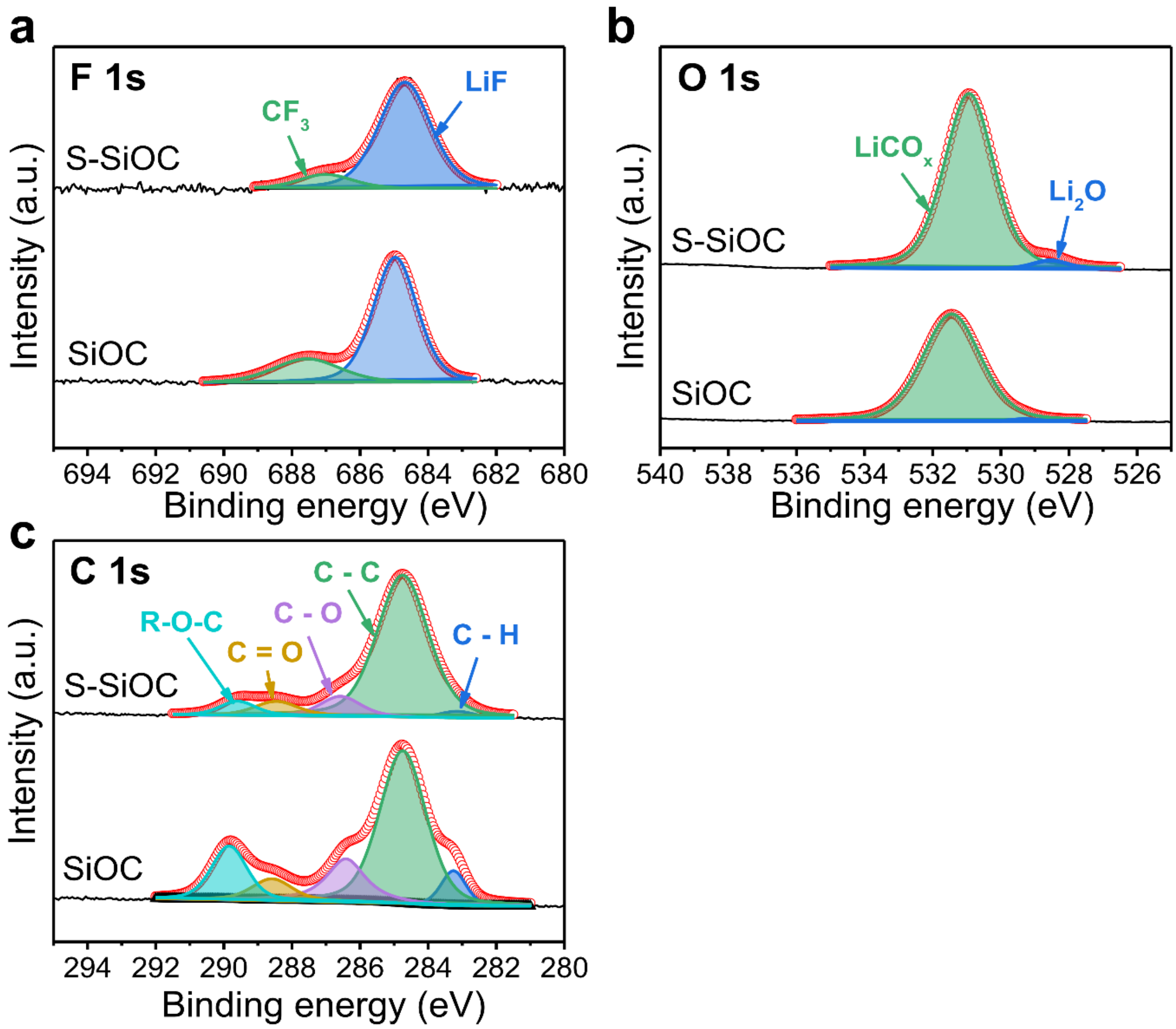

Fig. S6. XPS spectra of (a) F 1s, (b) O 1s, and (c) C 1s in SiOC and S-SiOC anode after initial cycle.

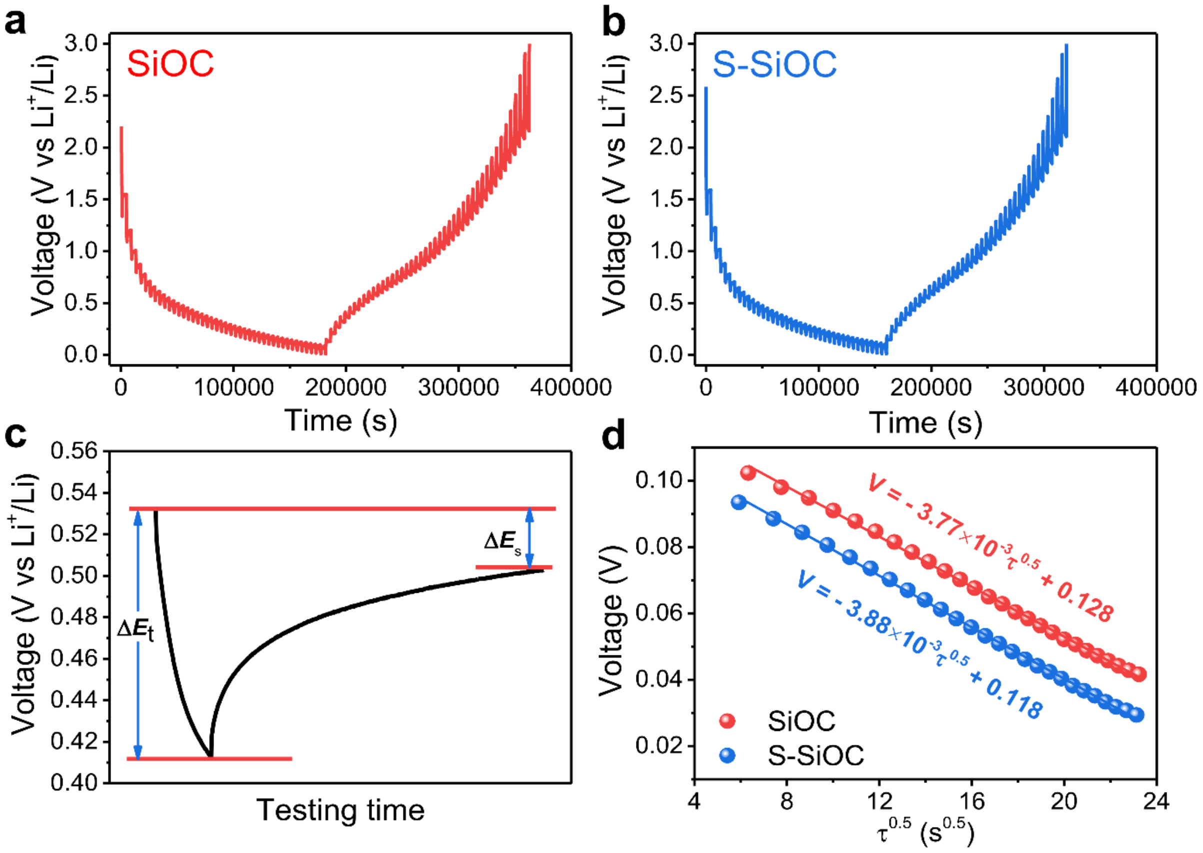

Fig. S7. Voltage profile of (a) SiOC and (b) S-SiOC during GITT analysis. (c) Demonstration of a single titration with ΔE_t, ΔE_s parameters. (d) Relationship between the voltage and square root of the pulse time at a single current pulse during GITT analysis of both electrodes.

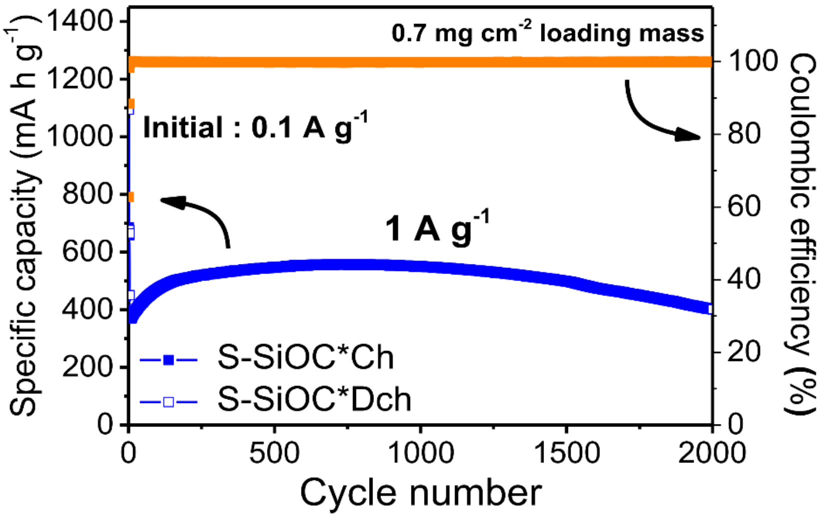

Fig. S8. Cycle performance and coulombic efficiency of S-SiOC electrode at 1 A g^{-1} for 2000 cycles (0.7 mg cm^{-2} of loading mass).

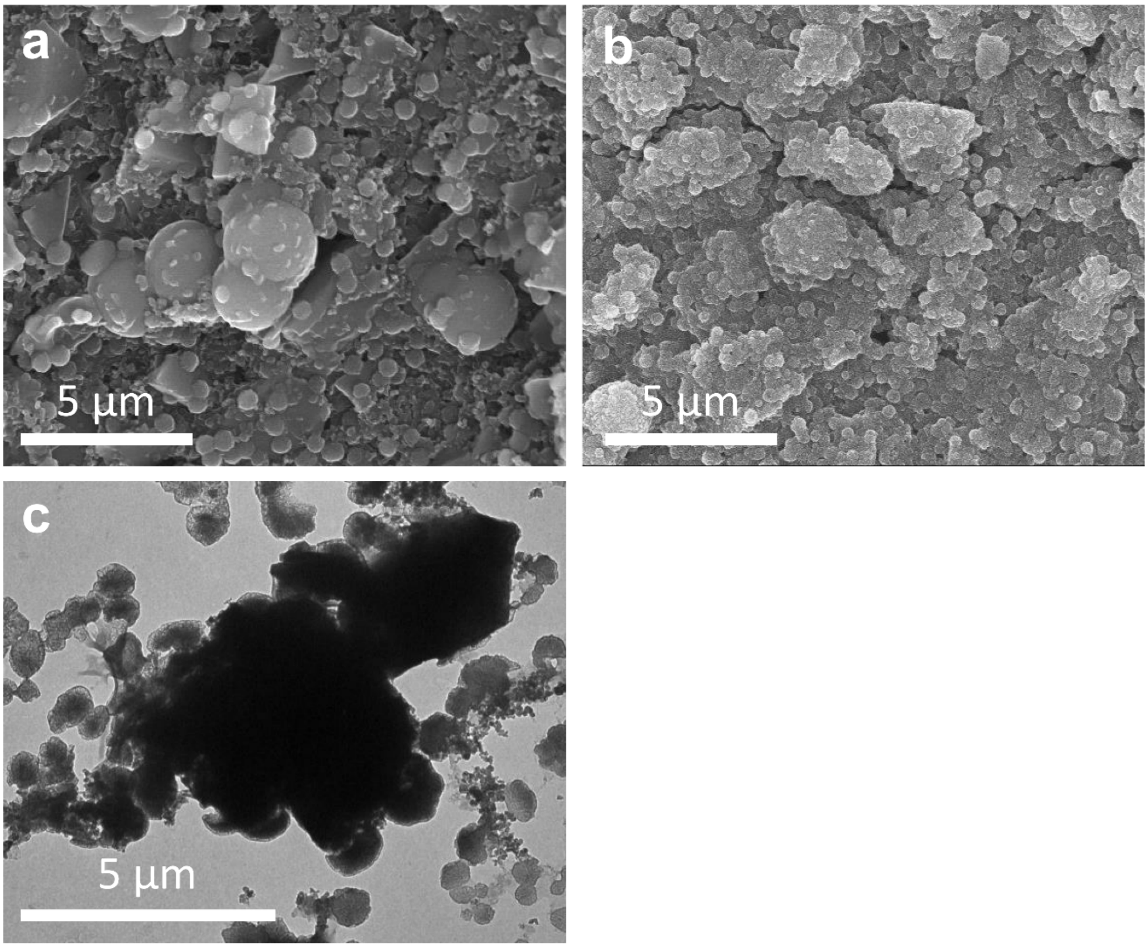

Fig. S9. SEM images of S-SiOC (a) before and (b) after cycling test. (c) TEM image of S-SiOC after cycling test.

Table S1. EIS fitting results of SiOC and S-SiOC electrodes.

Parameter	SiOC	S-SiOC
R_s / Ω	4.69	4.57
R_{SEI} / Ω	12.8	12.2
R_{ct} / Ω	87.2	43.0
W-R / Ω	0.00158	0.00461

Table S2. Comparison of electrochemical performances between the S-SiOC electrode and other reported SiOC-based electrodes.

Anode	Synthesis method	Initial specific capacity ($mAh\ g^{-1}$)	Initial Coulombic efficiency (%)	Cycle property	Ref.
S-SiOC	Facile one-pot pyrolysis (800°C for 5 hours)	1146 mAh g^{-1} at 0.1 A g^{-1}	69.9	89.2% retention after 2000 cycles at 1 A g^{-1}	This work
Onion-like pre-SiOC/C spheres	One-step injection pyrolysis (900°C for 30 min)	839.3 mAh g^{-1} at 0.1 A g^{-1}	78.4	82% retention after 500 cycles at 2 A g^{-1}	[1]
Divinylbenzene and polymethylsilsesquioxane coordinated SiOC (S-DVB-1)	One-step pyrolysis (1200°C for 1 hour)	1273.5 mAh g^{-1} at 0.1 A g^{-1}	64.38	Remaining capacity to 476 mAh g^{-1} after 500 cycles at 0.5 A g^{-1}	[2]
Rambutan-like vertical graphene coated hollow porous SiOC (Hp-SiOC@VG)	Hydrothermal (180°C for 12 hours) + pyrolysis (1000°C for 2 hours)	729 mAh g^{-1} at 0.1 A g^{-1}	75	98% retention after 600 cycles at 1 A g^{-1}	[3]
Intercalated SiOC/graphene composites	Pyrolysis (800°C for 4 hours)	965 mAh g^{-1} at 0.05 A g^{-1}	63	60% retention after 90 cycles	[4]
Nearly kilogram-scale proparation of SiOC composites	CVD method (1000°C)	988 mAh g^{-1} at 0.1 A g^{-1}	70	88% retention after 2000 cycles at 1	[5]

				A g^{-1}	
Amorphous polymer-derived SiOC (NGA-SiOC25)	Pyrolysis (900°C for 1 hour)	1116 mAh g^{-1} at 0.037 A g^{-1}	67.3	95% retention after 1000 cycles at 1.48 A g^{-1}	[6]
A SiOC bead mixture of vinyltrimethoxysilane with phenyltrimethoxysilane (Vi-Ph-SiOC)	MicroJet reactor technique + pyrolysis (1100°C for 3 hours)	922 mAh g^{-1} at 0.035 A g^{-1}	76.8	83% retention after 100 cycles at 0.07 A g^{-1}	[7]

References

[1] X. Lin, Y. Dong, X. Liu, X. Chen, A. Li, H. Song, In-situ pre-lithiated onion-like SiOC/C anode materials based on metallasilsesquioxanes for Li-ion batteries, Chem. Eng. J., 428 (2022) 132125.

[2] P. Wu, X. Guo, Z. Su, C. Liu, S. Chen, Z. Zheng, A. Liu, Preparation of silicon oxycarbide (SiOC) anodes for high performance Li-ion batteries using competitive relationship between crosslinking and polymeriz

[3] K. Li, G. Yuan, X. Liu, Y. Guo, R. Huang, H. Li, H. Zhang, Q. Jia, Z. Xie, S. Zhang, W. Lei, On the practical applicability of rambutan-like SiOC anode with Enhanced reaction kinetics for lithium-ion storage, Adv. Funct. Mater., 33 (2023) 2302348.

[4] Y. Ren, B. Yang, X. Huang, F. Chu, J. Qiu, J. Ding, Intercalated SiOC/graphene composites as anode material for Li-ion batteries, Solid State Ion., 278 (2015) 198-202.

[5] S. Fan, J. Zhang, S. Cui, L. Chen, X. Chen, J. Rang, W. Wang, Y. Liu, J.-T. Zhao, Large-

scale synthesis of SiOC composites for stable Li-ion battery anode and denrite-free Li metal deposition, Chem. Eng. J., 479 (2024) 147785.

[6] G. Shao, D.A.H. Hanaor, J. Wang, D. Kober, S. Li, X. Wang, X. Shen, M.F. Bekheet, A. Gurlo, Polymer-derived SiOC integrated with a graphene aerogel as a highly stable Li-ion battery anode, ACS Appl. Mater. Interfaces, 12 (2020) 46045-46056.

[7] B. Krüner, C. Odenwald, N. Jäckel, A. Tolosa, G. Kickelbick, V. Presser, Silicon oxycarbide beads from continuously produced polysilsesquioxane as stable anode material for lithium-ion batteries, ACS Appl. Energy Mater. 1 (2018) 2961-2970.
